# Impact of Entrepreneurial Education, Mindset, and Creativity on Entrepreneurial Intention: Mediating Role of Entrepreneurial Self-Efficacy

**DOI:** 10.3389/fpsyg.2021.724440

**Published:** 2021-08-23

**Authors:** Wang Jiatong, Majid Murad, Fu Bajun, Muhammad Shahid Tufail, Farhan Mirza, Muhammad Rafiq

**Affiliations:** ^1^College of International Business, Zhejiang Yuexiu University of Foreign Languages, Shaoxing, China; ^2^School of Management, Jiangsu University, Zhenjiang, China; ^3^School of Education, Shaoxing University, Shaoxing, China; ^4^Department of Economics and Business Administration, University of Education, Lahore, Pakistan; ^5^University of Management and Technology, Sialkot, Pakistan

**Keywords:** entrepreneurial education, entrepreneurial mindset, entrepreneurial self-efficacy, entrepreneurial intention, creativity, China

## Abstract

This study aimed to investigate the direct influence of entrepreneurial education, entrepreneurial mindset, and creativity on the entrepreneurial intention with the indirect role of entrepreneurial self-efficacy. This study applied the structural equation model technique using AMOS software to verify the hypothesis relationships. This study collected self-administered survey data from 365 university students of Jiangsu and Zhejiang province of China. The findings indicated that entrepreneurial education, entrepreneurial mindset, and creativity have a positive and significant influence on entrepreneurial intention. Moreover, results revealed that entrepreneurial self-efficacy partially mediates in the relationship between entrepreneurial education, entrepreneurial mindset, and creativity on entrepreneurial intention. Further implications and limitations are also discussed in this article.

## Introduction

The topic of entrepreneurship has received extensive attention among researchers over the past decades (Wadhwani et al., 2020). Entrepreneurship has become a dominant issue in developed and under-developed nations as well because it develops efforts in improving the economic welfare of the nation (Värlander et al., 2020; Yi, 2020). Entrepreneurship improves the economic and social growth of nations (Li et al., 2020a; Neneh, 2020). Previous studies have remarked that entrepreneurship education, entrepreneurial mindset, and creativity cultivate young talents and develop entrepreneurial intention among individuals to become entrepreneurs (Westhead and Solesvik, 2016; Hu et al., 2018; Pan et al., 2018; Jena, 2020) and argued that with an increasing number of university graduates, appropriate job searching has become a serious concern in the higher education system in China. According to Hu and Ye (2017) in developed countries, the success rate of entrepreneurship is more than 25% as compared to Chinese university graduates, who achieve only a 10% success rate due to a lack of entrepreneurial education, entrepreneurial mindset, and creativity. Most of the students prefer to start a job in a company rather than start their businesses. Therefore, the Chinese government has developed measures to alleviate the pressure of unemployment and provide suitable entrepreneurial platforms for students to become entrepreneurs.

Kalyoncuoǧlu et al. (2017) defined entrepreneurship education as associated with nurturing creative skills that can be applied in real life. Moreover, the entrepreneurial mindset has been recognized in providing success and failure among entrepreneurs in entrepreneurship research (Moore et al., 2021). Jena (2020) argued that entrepreneurial mindset is associated with the profound cognitive phenomena that reflect the inimitable commitment of entrepreneurial activities (Saptono et al., 2020). The term creativity is defined as the creation of new and useful ideas (Entrialgo and Iglesias, 2020). Previous scholars indicated that there are several supportive dimensions such as entrepreneurial education (Barba-Sánchez and Atienza-Sahuquillo, 2018), entrepreneurial passion (Karimi, 2020), entrepreneurial orientation (Cho and Lee, 2018), entrepreneurial self-efficacy (Schmutzler et al., 2019), and entrepreneurial mindset (Cui et al., 2019) and creativity are associated with the development of an entrepreneurial intention for new business startups. Therefore, the direct relationship of entrepreneurial education, entrepreneurial mindset, and creativity are less studied in the context of Chinese student entrepreneurial intention. Consequently, the objective of this study is to identify the influence of these factors on entrepreneurial intention among Chinese students.

The social cognitive theory proposed by Bandura (1992) outlines that entrepreneurial education improves an individual's self-efficacy. It allows individual to have an opportunity in entrepreneurship tasks such as identifying an opportunity, making business feasibility, and implementing a business plan. In line with the entrepreneurship research in the context of developed nations, the role of entrepreneurial education, entrepreneurial mindset, and creativity in entrepreneurship have been discussed by several researchers (Shi et al., 2020; Anjum et al., 2021). Some studies have outlined that individuals with a high level of entrepreneurial education, entrepreneurial mindset, and creativity are more prone to start their businesses (Hu and Ye, 2017; Handayati et al., 2020). Additionally, scholars believed that the understanding of entrepreneurial self-efficacy is essential, especially on how to start, manage and develop a new business (Chien-Chi et al., 2020; Lingappa et al., 2020). Thus, individuals that perceived a high level of self-efficacy will lead to greater cognitive minds. Neneh (2020) pointed out that self-efficacy is a social-cognitive process that elaborates the cognitive mindset of individuals in the shape of entrepreneurial intention and entrepreneurial behavior.

This study provides four main contributions to literature on entrepreneurship. First, existing studies have been focused on entrepreneurial traits such as family business (Douglas et al., 2021), big five personality traits (Bazkiaei et al., 2020), the dark side of personality traits (Cai et al., 2021), entrepreneurial self-efficacy (Ceresia and Mendola, 2020), and entrepreneurial alertness (Urban, 2020) to determine the entrepreneurial intention of the student. Second, Wardana et al. (2020) argued that there is a lack of study into an entrepreneurial education and entrepreneurial mindset on entrepreneurial intention; most of the previous studies have been investigated on entrepreneurship education and entrepreneurial mindset in the context of Europe (Boldureanu et al., 2020), America (Barnard et al., 2019), Africa (Puni et al., 2018), Malaysia (Shamsudin et al., 2017), and India (Jena, 2020), while little attention has been paid by scholars to the context of China.

Third, a recent study by Handayati et al. (2020) examined entrepreneurship education to assess the entrepreneurial mindset of the vocational student in Indonesia. This study provides an extension to the model by Handayati et al. (2020) and Jena (2020) using entrepreneurial education, entrepreneurial mindset, and creativity as independent variables and taking entrepreneurial self-efficacy as a mediator to predict the entrepreneurial intention in Chinese students. Fourth, this study contributes to the social cognitive theory by Bandura (1985) that helps explain the individual self-efficacy, which helps entrepreneurs develop. Numerous researchers have discussed the positive influence of self-efficacy in social psychological research (Alonso et al., 2020; Mozahem and Adlouni, 2021). Meanwhile, many researchers discuss the positive mediating influence of entrepreneurial self-efficacy on entrepreneurial intention (Fernando and Nishantha, 2019; Burnette et al., 2020). Thus, this study attempts to identify the mediating role of entrepreneurial self-efficacy in explaining entrepreneurial education, entrepreneurial mindset, and creativity, which ultimately influence an individual when starting a new business.

Based on the above-mentioned research impetus, this study aims to address these research gaps in the conceptualization of entrepreneurial education, entrepreneurial mindset, creativity, entrepreneurial self-efficacy, and entrepreneurial intention. Thus, within the developed research model the study addresses the following questions:

**RQ1**. What is the influence of entrepreneurial education, entrepreneurial mindset, and creativity on entrepreneurial intention among Chinese students?
**RQ2**. Does entrepreneurial self-efficacy mediate the relationship between entrepreneurial education, entrepreneurial mindset, and creativity in entrepreneurial intention among Chinese students?

The present study discusses the development of theory and the hypothesis, before describing the methods used, before moving onto the the results and discussion, implications and limitations, and conclusions.

## Theory and Hypothesis Development

Social cognitive theory refers to a learning theory that focuses on observational learning of the individual, modeling, and self-efficacy (Beauchamp et al., 2019). This theory remarked that individuals are inclined to pursue their objectives if they consider their skills and abilities are capable of accomplishing the desired outcomes (Lim et al., 2020; Wu et al., 2020). Entrepreneurial education helps individuals to enhance their social cognition, continually regulate their thoughts and make their entrepreneurial actions more directional, logical, and significant. This study employs the social cognitive theory to assess how students with a high level of entrepreneurial education, entrepreneurial mindset, and creativity improve their ability to develop entrepreneurial self-efficacy which in turn affects entrepreneurial intention (Yuan et al., 2020).

Furthermore, previous research believed that general education emphasizes the overall progress of individuals and the entrepreneurial program lays the foundation for the overall growth of the skills of an individual (Liguori et al., 2018). From the perspective of social learning theory, individuals are encouraged to learn through different multi-level channels and enhance their skills and knowledge to become entrepreneurs (Oo et al., 2018). Thus, entrepreneurial education, entrepreneurial mindset, and creativity improve the learning environment of individuals and enhance their confidence level that will be able to solve new and unexpected issues regarding the new business development (Chia and Liang, 2016).

### Entrepreneurial Education and Entrepreneurial Mindset

Prior studies argued that entrepreneurial education has a positive relationship with the entrepreneurial mindset (Pfeifer et al., 2016; Karyaningsih et al., 2020). Entrepreneurial education defied as a learning activity that is associated with the improvement of knowledge, abilities, skills, and personal character regarding entrepreneurship education (Cui et al., 2019; Yuan and Wu, 2020). Moreover, an entrepreneurial mindset is defined as a feeling or propensity to provide a creative and innovative thinking ability (Günzel-Jensen et al., 2017). Prior studies discussed the idea of entrepreneurial mindset in the field of psychology, especially in personality psychology research, and found that entrepreneurial mindset is positively related to self-capability (Zupan et al., 2018; Morris and Tucker, 2021). Furthermore, numerous researchers focused on the entrepreneurial mindset and its factors such as knowledge, skills, abilities, creative ideas, and attitude toward entrepreneurship (Green et al., 2020; Rodriguez and Lieber, 2020; Saptono et al., 2020) believed that the entrepreneurial mindset is associated with individual attitude and entrepreneurial action.

Wardana et al. (2020) discuss how entrepreneurial education enables people to have capability, providing them with understating about how to identify opportunities and develop their attitude toward entrepreneurship. Handayati et al. (2020) remarked that entrepreneurship education promotes the entrepreneurial mindset of individuals from two perspectives. First, entrepreneurial education assists individuals to develop a culture and intensely understand entrepreneurship. Second, entrepreneurial education creates awareness among individuals to get more experience to start a new business (Barnard et al., 2019). Therefore, we believed that individuals with a high level of entrepreneurial education are more likely to have an entrepreneurial mindset, which enables them to become entrepreneurs and propose the following hypothesis:

**H1:** Entrepreneurial education is positively related to the entrepreneurial mindset.

### Entrepreneurial Education and Entrepreneurial Intention

Entrepreneurship education is related to the ability of actions of the individual in favor of knowledge and abilities (Liu et al., 2019). Previous research believed that entrepreneurial education has an important role in improving the skills of the individual that stimulates business activities (Sun et al., 2017). Yang (2014) remarked that entrepreneurial education has two key features. First, through entrepreneurial learning actions, it facilitates individuals to transfer knowledge, skills, and share experience of entrepreneurship. Second, entrepreneurial education through field studies motivates individuals to be successful person in the future. Moreover, a study argued that entrepreneurial education provides help to individuals in achieving entrepreneurial intention through social networks and the experience of successful entrepreneurs (Vodǎ and Florea, 2019).

Entrepreneurial education assists individuals to obtain minimal resources through appropriate knowledge sharing and information transfer. Therefore, individuals who show interest in entrepreneurial learning are more likely to engage with peers and fellows and promote the entrepreneurial image (Nowiński et al., 2019). The role of entrepreneurial education for entrepreneurial intentions can be demonstrated by understanding business education (Turner and Gianiodis, 2018). Entrepreneurship education allows individuals to improve their mindfulness and entrepreneurship intention for a career path to work (Kalyoncuoǧlu et al., 2017). The basic function of entrepreneurial education focuses on the enrichment of knowledge, skill, and attitude toward entrepreneurship. Thus, based on the existing studies we argued that individuals who perceived a high level of entrepreneurial education are more likely to pursue a career in entrepreneurship. Hence, we hypothesized that:

**H2:** Entrepreneurial education is positively related to entrepreneurial intention.

### Entrepreneurial Mindset and Entrepreneurial Intention

According to Hsu et al. (2019) entrepreneurship intention is defined as a self-acknowledged belief to start a new career. Moreover, studies have asserted that entrepreneurial intention is associated with the identification, evaluation, and exploitation of new opportunities with the help of planning, organizing, processes, and raw materials (Miranda et al., 2017; Barba-Sánchez and Atienza-Sahuquillo, 2018). Previous studies revealed that entrepreneurial mindset is positively related to entrepreneurial intention (Cui et al., 2019; Handayati et al., 2020). Entrepreneurial mindset refers to an individual commitment toward entrepreneurial activities (Kuratko et al., 2020). An entrepreneurial mindset contains an inclination of the individual with the combination of risk-taking, need for achievement, and passion to start a new business as well as develop, plan, and organize projects to achieve entrepreneurial goals (Bosman and Fernhaber, 2019).

Handayati et al. (2020) conducted a study on the entrepreneurial minds of vocational students in Indonesia and found that entrepreneurial mindset had a positive and significant influence on entrepreneurial intention. Furthermore, Wardana et al. (2020) examined a study on the entrepreneurial mindset and entrepreneurial intention using a 390 university student sample and found that entrepreneurial mindset positively related to entrepreneurial intention. Meanwhile, Jung and Lee (2020) investigated a study on entrepreneurial minds of college students to predict their entrepreneurial intention in South Korea, and results show that entrepreneurial traits such as innovativeness, autonomy, and pro-activeness were positively developed the entrepreneurial mindset of students to become entrepreneurs.

The entrepreneurial mindset develops over time and needs to be used regularly (Aima et al., 2020). Therefore, individuals must make their minds more efficient during daily life and pay more attention to opportunities (Kaffka and Krueger, 2018). Based upon these past studies, we believe that individuals with entrepreneurial mindsets more actively participate in entrepreneurial activities than other individuals. Consequently, we hypothesized that:

**H3:** Entrepreneurial mindset is positively related to entrepreneurial intention.

### Creativity and Entrepreneurial Intention

Creativity is an essential feature of individual cognitive processing and can produce new and useful ideas through appropriate information and knowledge (Zampetakis and Moustakis, 2006). According to Rodrigues et al. (2019), creativity is defined as the ability and skill that people hold. Prior researchers discussed that creativity is particularly essential for entrepreneurial activities and entrepreneurship itself is a creative activity (Kumar and Shukla, 2019; Shi et al., 2020). Similarly, Hu et al. (2018) conducted a study using creativity and entrepreneurial alertness and found significant results in the context of university students in China. Furthermore, a recent study investigated a sample of 390 university students in Pakistan and found the significant impact of creativity in the relationship between entrepreneurial passion and entrepreneurial intention (Murad et al., 2021).

Zampetakis et al. (2011) studied the relationship between creativity and entrepreneurial intention using undergraduate business students and found that individuals with a higher level of creativity are more likely to become entrepreneurs. Chia and Liang (2016) conducted a study to examine the impact of creativity on the entrepreneurial intention of university tourism students in Taiwan and remarked that students who perceived high creativity are more prone to start a new business. Shi et al. (2020) studied the relationship between creativity and the theory of planned behavior (TPB) on entrepreneurial intention using a survey of 523 university students in China and found that individuals with a high level of creativity can obtain a positive attitude and high self-belief in entrepreneurial activities.

Additionally, Miranda et al. (2017) used a 1,178 Spanish university student sample to identify the influence of attitude, subjective norms, and perceived behavioral control on creativity and entrepreneurial intention and found that individuals with high creative minds are more likely to engage in entrepreneurial activities. Based on the above literature, most of the previous studies found a positive correlation between creativity and entrepreneurial intention. Thus, we believed that creativity will positively lead toward entrepreneurial intention.

**H4:** Creativity is positively related to entrepreneurial intention.

### The Mediating Role of Self-Efficacy

Self-efficacy is defined as individual self-belief to attain goal-oriented tasks (Barbaranelli et al., 2019). Self-efficacy is also associated with the inclination of individuals to achieve their personal goals (Newman et al., 2019). The concept of self-efficacy is derived from social cognitive theory. This theory was proposed by Bandura (1985) which demonstrated that individual behavior is developed by numerous activities such as interpersonal, involvement, and circumstance. The relationship between these activities can form confidence in an individual in encompassing the ability to manage certain behaviors and their expectations of behavioral results (Nowiński et al., 2019).

A prior study discussed that self-efficacy is an influential factor in explaining individual entrepreneurial intention and behavior (Schmutzler et al., 2019). Moreover, an increasing number of researches in entrepreneurial intention/behavioral models found the significant mediating role of self-efficacy as a direct and indirect variable in the field of entrepreneurship and social psychology (Newman et al., 2019; Li et al., 2020b). McGee and Peterson (2019) revealed that self-efficacy is the essential factor that affects the behavior of an individual through the cognitive process, objective setting, and result expectations. Furthermore, scholars argued that entrepreneurs with extraordinary self-efficacy for a particular task are more likely prone to entrepreneurial activities rather than other entrepreneurs who have less self-efficacy (Şahin et al., 2019; Urban, 2020).

Burnette et al. (2020) believed that self-efficacy explains the cognition process, develops creative thinking, and helps individuals in the decision-making process to start a new business. In the cognitive process, previous scholars paid much attention to individual creative thinking toward new business startups (Schmitt et al., 2018; Hsu et al., 2019). Kumar and Shukla (2019) examined the direct influence of creativity and proactive personality with the mediating role of entrepreneurial self-efficacy to measure entrepreneurial intention among university students in India and found that creativity positively leads toward entrepreneurial self-efficacy and entrepreneurial intention. Thus, individuals with a higher level of entrepreneurial self-efficacy are more likely to perceive higher entrepreneurial education, entrepreneurial mindset, and creativity. Hence, we proposed the following hypotheses:

**H5:** Entrepreneurial self-efficacy is positively related to entrepreneurial intention.
**H5a:** The relationship between entrepreneurial education and entrepreneurial intention will be mediated by entrepreneurial self-efficacy.
**H5b:** The relationship between entrepreneurial mindset and entrepreneurial intention will be mediated by entrepreneurial self-efficacy.
**H5c:** The relationship between creativity and entrepreneurial intention will be mediated by entrepreneurial self-efficacy.

**Figure 1:**
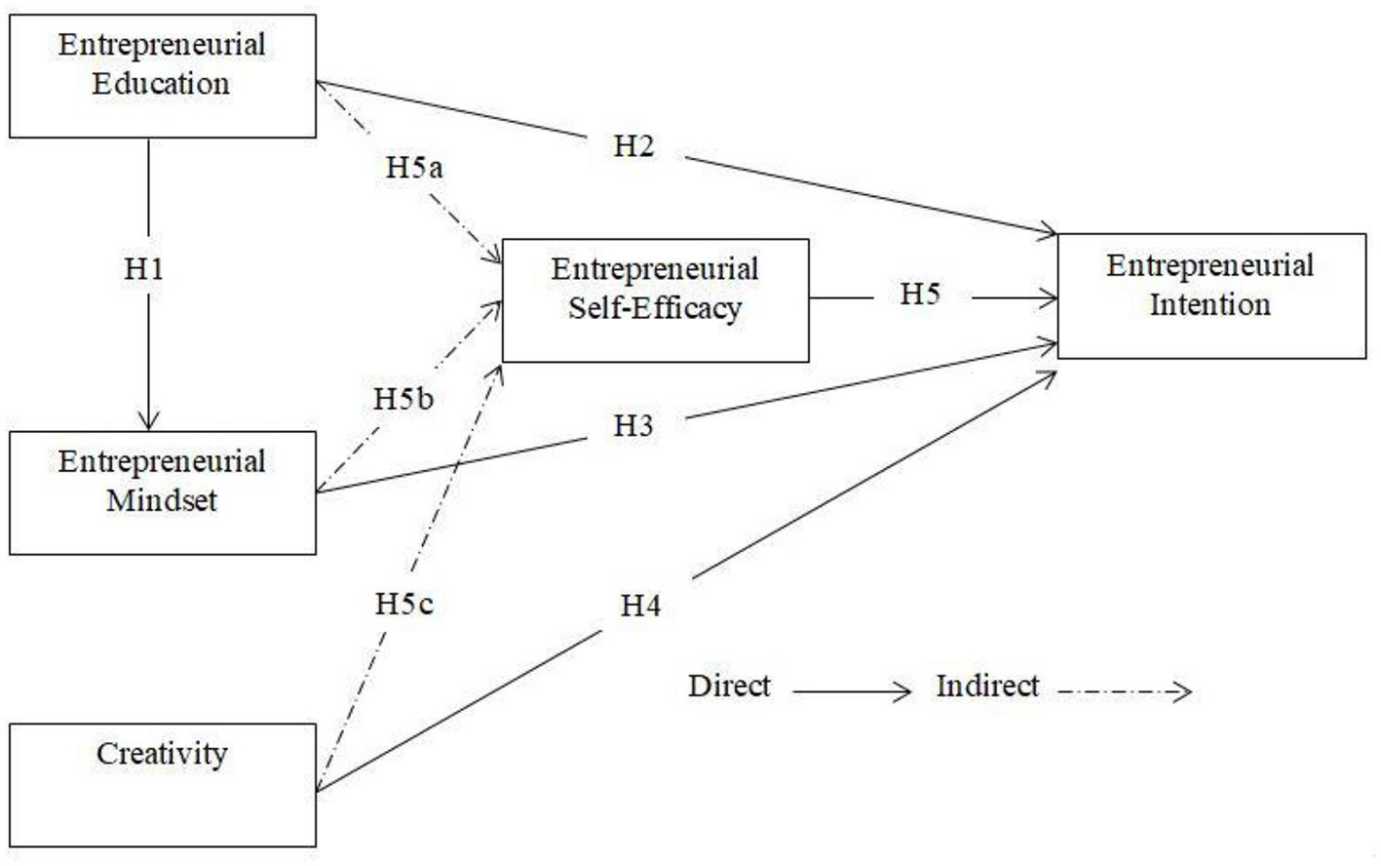
Conceptual model.

The conceptual model depicting the relationships and hypothesis is given in Figure 1.

## Methods

### Pilot Survey and Sampling Technique

A pilot test was applied by distributing 100 questionnaires to business students at Jiangsu and Zhejiang universities in China. We received 75 effective responses with a participation rate of 75%. Based on the pilot survey feedback, the reliability and validity of the measurement constructs were acceptable. The target population of this study includes all enrolled university students of Jiangsu and Zhejiang provinces in China. This study focused on the entrepreneurial intention of the business student because there is a growing trend in the field of entrepreneurship that university students were more involved in business startups (Li et al., 2020b; Neneh, 2020). Moreover, this study applied a convenience sampling technique and a survey was conducted from February 05, 2021, to April 30, 2021. The original draft of the questionnaires was in English language and translation was checked by using the translation and back-translation process through the two language experts who have a good command of Chinese and English language. Furthermore, we distributed 450 paper and pencil questionnaires among respondents, and 380 received with a participation rate of 84%. We discarded 15 questionnaires due to incomplete forms of responses, thus, the final response size included 365 valid questionnaires. The participation of the respondents was voluntary and students who engaged in this survey were announced for their anonymity.

Among the valid responses (55.1%) were male and (44.9%) were female. In terms of age groups were 18–25 (44.1%), 26–35 (32.9%), 36–45 (17.5%), and 45–above (5.5%). In terms of major distribution school of management accounted for (43%), the school of finance accounted for (34.2%), the school of public administration accounted for (20%), and the school of economics accounted for (2.7%). There were (47.7%) undergraduate students (29.6%), masters students (20%), diploma and other, and (2.7%) PhD students. Also, (60.3%) came from a family with an entrepreneurial background, and (39.7%) were not belonging to the entrepreneurial family background.

Additionally, Harman single factor test was performed on the data. According to Harman methodology, all the factors are merged in the factor analysis and the first factor explained more than 50% of the total variance that means there is an issue of common method bias in the data. The results from factor analysis show that the first factor explained 30.43% of the total variance. Therefore, there is no issue of common method bias in this study (Podsakoff, 2003).

### Measures

This study adopted measurement scales that had been tested and validated by the previous researchers. We used a 5-point Likert scale rating from 1 strongly disagree to 5 strongly agree and evaluated the responses of students.

#### Entrepreneurial Education

To assess entrepreneurial education, we used six items from the prior study by Wardana et al. (2020). This scale was used by previous researchers to predict the entrepreneurial education of students (Handayati et al., 2020). A sample item “I believe that entrepreneurial education in school drives business students to be entrepreneurs.” The Cronbach's α for entrepreneurial education was 0.936.

#### Entrepreneurial Mindset

To measure the entrepreneurial mindset, we used six measurement constructs from the previous study by Wardana et al. (2020). A sample item “I have seen time allocation for entrepreneurial matters.” The Cronbach's α for entrepreneurial mindset was 0.905.

#### Entrepreneurial Self-Efficacy

To evaluate entrepreneurial self-efficacy, we used four items from the study by Zhao et al. (2005). This scale was widely used by prior scholars to assess entrepreneurial self-efficacy (Li et al., 2020b). A sample item “I am convinced that I can successfully create new products.” The Cronbach's α for entrepreneurial self-efficacy was 0.918.

#### Entrepreneurial Intention

To measure the entrepreneurial intention of business students, we used six items from the study by Liñán et al. (2011). This scale was applied by several researchers to evaluate the student entrepreneurial intention (Mahmood et al., 2019; Neneh, 2019). A sample item “I am determined to start a new business in the future.” The Cronbach's α for the entrepreneurial intention was 0.939.

#### Creativity

To evaluate creativity, we used six measurement constructs from the study by Biraglia and Kadile (2017). This scale was also used by previous researchers (Kumar and Shukla, 2019; Murad et al., 2021). A sample item “I often have new and innovative ideas.” The Cronbach's α for creativity was 0.922.

## Results

### Measurement Model

**Figure 2:**
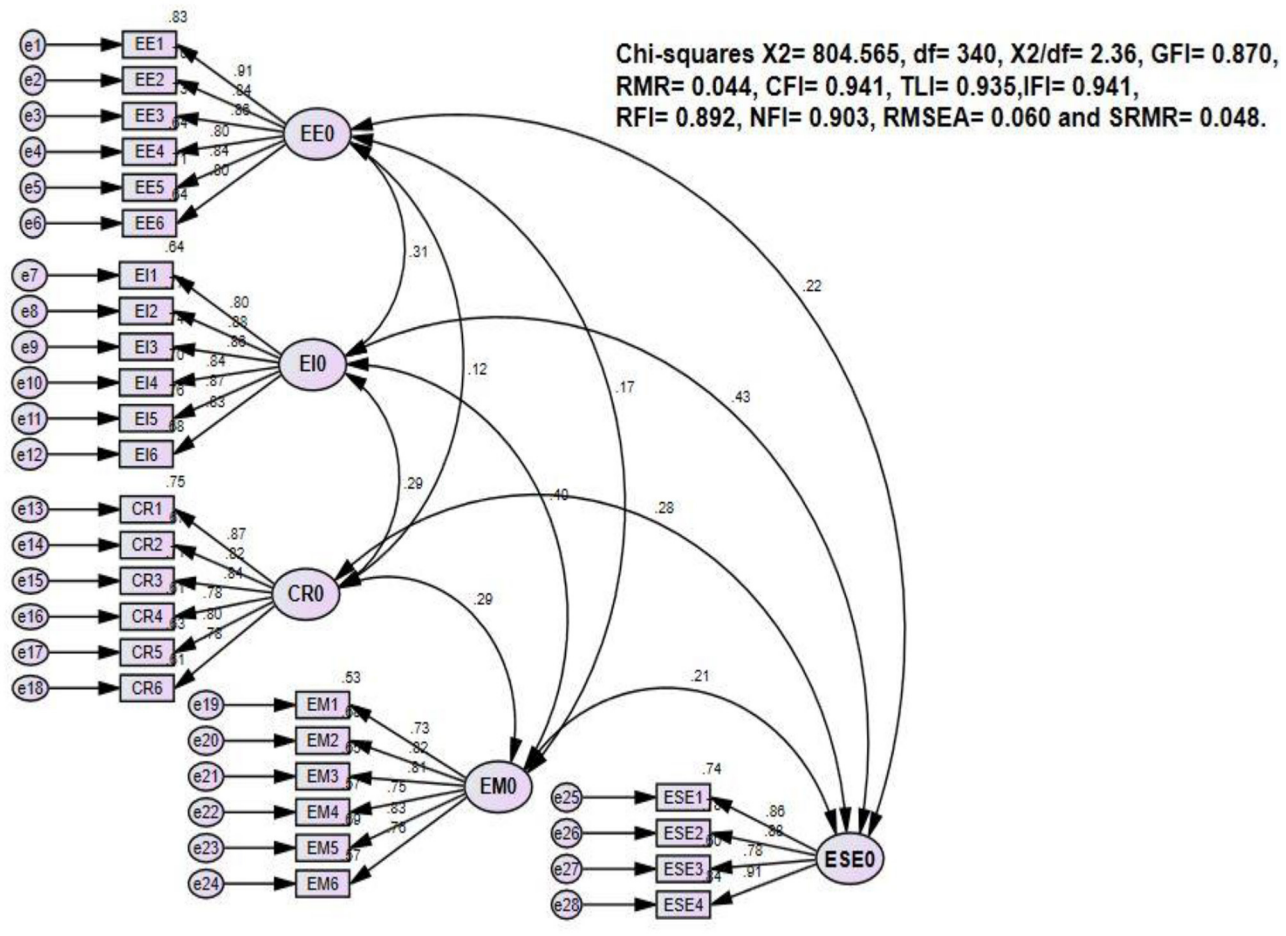
Confirmatory factor analysis (CFA) results.

The confirmatory factor analysis (CFA) was performed by utilization of the AMOS software and findings were presented in Table 1 and Figure 2). Moreover, Table 2 results show that five measurement constructs have satisfactory reliability results because all the values of Cronbach's α surpassed 0.70 and the composite reliability ranged from 0.908 to 0.941 exceeded the recommended benchmark of 0.60 (Bagozzi et al., 1991). Also, about the validity test, all the measured items factor loadings ranged from 0.725 to 0.915 (all *p* < 0.001). The values of the average variance extracted were satisfactory and ranged from 0.613 to 0.738 (as shown in Table 2).

**Table 1:** Confirmatory factor analysis (CFA).

**Constructs**	**Items**	**Measures**	**Std. β**	**S.E**	***Z***	***p***
**Creativity**						
	CR 1	I often come up with creative solutions to problems.	0.866	-	-	-
	CR 2	I am good at providing a fresh approach to problems.	0.817	0.043	19.691	^***^
	CR 3	I often come up with new and practical ideas.	0.844	0.045	20.793	^***^
	CR 4	I often have new and innovative ideas.	0.784	0.043	18.371	^***^
	CR 5	I am good at generating creative ideas.	0.795	0.046	18.826	^***^
	CR 6	I often promote and champion ideas to others.	0.784	0.046	18.376	^***^
**Entrepreneurial intention**						
	EI 1	I am ready to do anything to be an entrepreneur.	0.803	-	-	-
	EI 2	My professional goal is to become an entrepreneur.	0.877	0.060	19.818	^***^
	EI 3	I will make every effort to start and run my own firm.	0.863	0.061	19.341	^***^
	EI 4	I am determined to create a firm in the future.	0.839	0.062	18.582	^***^
	EI 5	I have the firm intention to start a firm someday.	0.874	0.060	19.731	^***^
	EI 6	I have a strong intention to start a business someday.	0.826	0.063	18.190	^***^
**Entrepreneurial education**						
	EE 1	The entrepreneurial education model in the formal setting promotes the creative ideas.	0.909	-	-	-
	EE 2	The learning model in the classroom provides the required knowledge toward entrepreneurship.	0.839	0.039	22.807	^***^
	EE 3	The education in school drives skill and ability related to entrepreneurship.	0.855	0.039	23.807	^***^
	EE 4	The education activities incorporate entrepreneurship matter and allow opportunities to students to begin a business.	0.802	0.040	20.802	^***^
	EE 5	I think that entrepreneurship occasion could be enlarge through education activities.	0.841	0.041	22.960	^***^
	EE 6	I believe that entrepreneurial education in school drives vocational students to be entrepreneurs.	0.800	0.040	20.700	^***^
**Entrepreneurial mindset**						
	EM 1	I have thought from both sides (opportunities or challenges) reactions incorporating with the entrepreneurial activities.	0.725	-	-	-
	EM 2	I have seen time allocation for entrepreneurial matters.	0.822	0.077	15.199	^***^
	EM 3	I have deliberated the financial chances to be engaged in the entrepreneurial activities.	0.806	0.076	14.909	^***^
	EM 4	I have evaluated for both opportunities and challenges linked with entrepreneurial activities.	0.752	0.077	13.889	^***^
	EM 5	I have decided toward ideas for business opportunity in the entrepreneurial activities.	0.829	0.072	15.327	^***^
	EM 6	I have disserted whether it is beneficial for me to be engaged in the entrepreneurial activities.	0.757	0.075	13.988	^***^
**Entrepreneurial self-efficacy**						
	ESE 1	I am convinced that I can successfully discover new business opportunities.	0.860	-	-	-
	ESE 2	I am convinced that I can successfully create new products.	0.881	0.045	22.300	^***^
	ESE 3	I am convinced that I can think creatively.	0.775	0.052	17.980	^***^
	ESE 4	I am convinced that I can successfully commercialize ideas.	0.915	0.044	23.700	^***^

*** *Significant (p < 0.001)*.

**Table 2:** Reliability and validity analysis.

	**Cronbach's α**	**AVE**	**Composite reliability**	**EE**	**EI**	**CR**	**EM**	**ESE**
EE	0.936	0.709	0.941	**0.842**				
EI	0.939	0.718	0.941	0.310	**0.847**			
CR	0.922	0.665	0.925	0.121	0.293	**0.816**		
EM	0.905	0.613	0.908	0.175	0.399	0.290	**0.783**	
ESE	0.918	0.738	0.928	0.218	0.427	0.279	0.208	**0.859**

*EE, Entrepreneurial education; EI, Entrepreneurial intention; CR, Creativity; EM, Entrepreneurial mindset; ESE, Entrepreneurial self-efficacy*.

*Values with diagonals are the square root of the AVE*.

*Values under diagonals are correlations (p < 0.001)*.

Furthermore, to assess the discriminant validity, we used criteria given by Fornell and Larcker (1981). Table 2 shows that the measurement model has satisfactory results because the square roots of AVE were greater than the values of its corresponding rows and columns. For the goodness-of-fit index, the results were presented as follow: *X*^2^ = 804.565, *X*^2^/df = 2.36, GFI = 0.870, CFI = 0.941, NFI = 0.903, RMSEA = 0.060, and SRMR = 0.048. Thus, all the values of measurement model constructs were acceptable and allowed the analysis of the structural model.

### Structural Model

**Figure 3:**
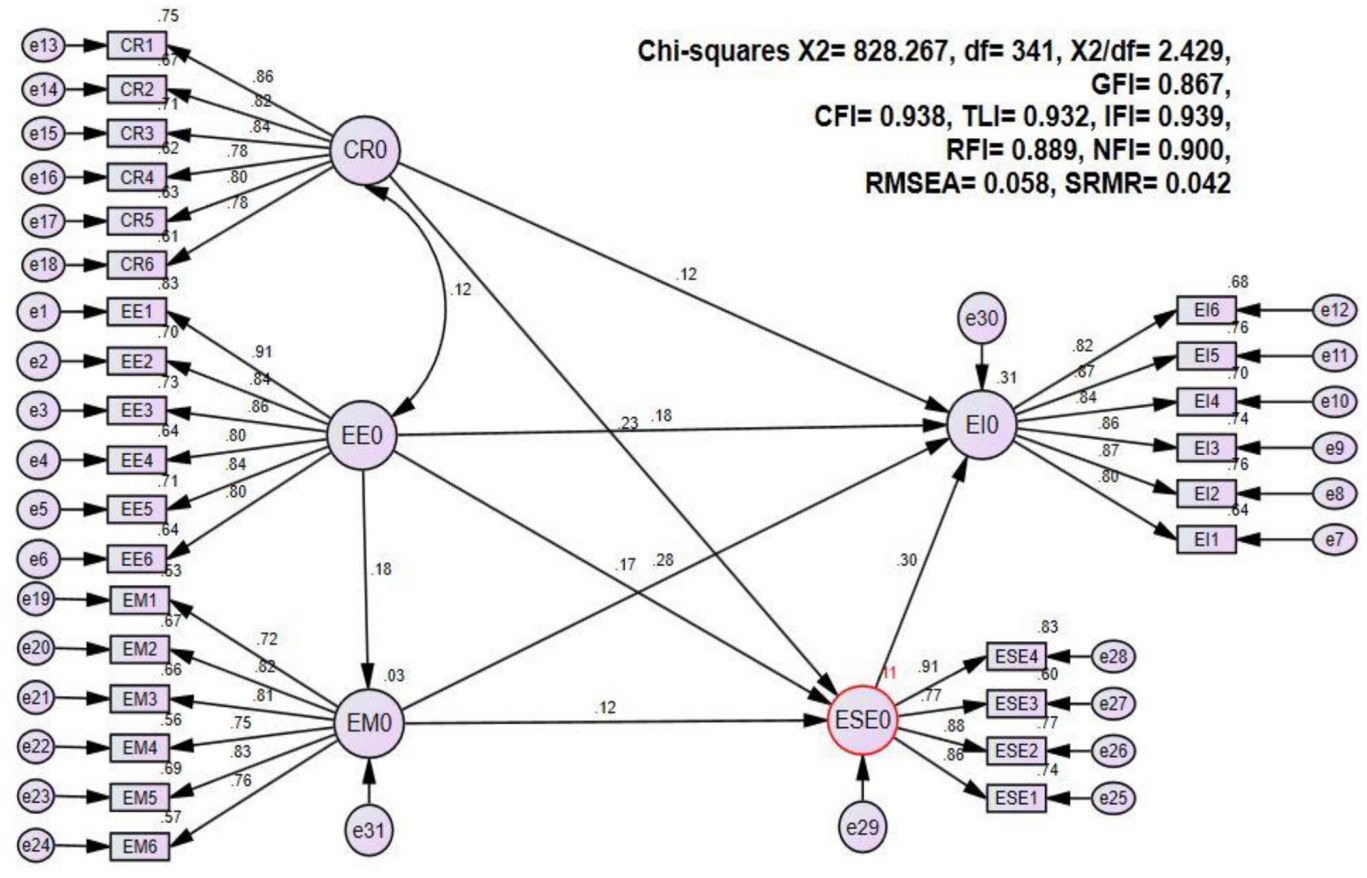
Structural model.

The structural model was assessed through the 5,000 bootstrapping method using the Amos software package. The findings of the structural model are expressed in Figure 3 which presented that all the results are satisfactory. Moreover, we tested the proposed hypotheses and the findings are shown in Table 3 and Figure 3. We found that entrepreneurial education had a direct positive and significant effect on entrepreneurial mindset (*β* = 0.177, critical ratio = 3.113, *p* < 0.002). Therefore, H1 was accepted. Furthermore, results indicate that entrepreneurial education had a direct positive and significant influence on entrepreneurial intention (*β* = 0.185, critical ratio = 3.671, *p* < 0.001). Thus, H2 was supported.

**Table 3:** Direct effects.

**Hypotheses**	**Relationships**	**Un-standardized estimates**	**S.E**	**Critical ratio**	***p*-value**	**Standardized estimates**
H1	EE → EM	0.150	0.048	3.113	0.002	0.177^**^
H2	EE → EI	0.191	0.052	3.671	0.001	0.185^**^
H3	EM → EI	0.337	0.064	5.240	0.001	0.276^**^
H4	CR → EI	0.112	0.049	2.300	0.001	0.116^**^
H5	ESE → EI	0.255	0.045	5.665	0.021	0.302^*^

*EE, Entrepreneurial education; EI, Entrepreneurial intention; CR, Creativity; EM, Entrepreneurial mindset; ESE, Entrepreneurial self-efficacy; S.E, Standard error*.

*Significant*

* *p < 0.05*,

** *p < 0.001*.

Meanwhile, results show that entrepreneurial mindset had a direct positive and significant impact on entrepreneurial intention (*β* = 0.276, critical ratio = 5.240, *p* < 0.001). Consequently, H3 was accepted. Additionally, findings illustrate that creativity had a direct positive and significant effect on entrepreneurial intention (*β* = 0.116, critical ratio = 2.300, *p* < 0.021). Moreover, H4 was supported. Lastly, we found that entrepreneurial self-efficacy had a direct positive and significant impact on entrepreneurial intention (β = 0.302, critical ratio = 5.665, *p* < 0.001). Hence, H5 was accepted.

To test the indirect effect of entrepreneurial self-efficacy in the relationship between entrepreneurial education, entrepreneurial mindset, and creativity, the bootstrap test was applied at a 95% confidence interval with 5,000 bootstrap samples. We followed the recommendations by Preacher and Hayes (2008) to calculate the confidence interval of the lower and upper bounds of bias-corrected percentile and percentile method to analyze whether the indirect effect was significant or not. Table 4 presents the results which indicate that entrepreneurial self-efficacy had a positive and significant indirect effect in the relationship between entrepreneurial education (*β* = 0.107, *p* < 0.001), entrepreneurial mindset (*β* = 0.035, *p* < 0.001) and creativity (*β* = 0.069, *p* < 0.001) on entrepreneurial intention. Thus, H5a, H5b, and H5c were also accepted.

**Table 4:** Indirect effects.

**Path coefficients and hypotheses**	**Std. estimations**	**Bootstrapping 5,000 samples with a 95% confidence interval**		***P-Value***
		**Bias-correlated percentile Lower and Upper**	**Percentile Lower and Upper**	
**H5a**				
**Standardized direct effects**				
EE → EI	0.185^**^	0.069, 0.324	0.053, 0.305	0.001
**Standardized indirect effects**				
EE → ESE → EI	0.107^**^	0.052, 0.167	0.052, 0.167	0.001
Standardized total effects	0.292^**^	0.161, 0.425	0.148, 0.413	0.001
**H5b**				
**Standardized direct effects**				
EM → EI	0.276^**^	0.169, 0.389	0.168, 0.389	0.001
**Standardized indirect effects**				
EM → ESE → EI	0.035^**^	0.01, 0.084	0.001, 0.083	0.001
Standardized total effects	0.312^**^	0.206, 0.415	0.206, 0.416	0.001
**H5c**				
**Standardized direct effects**				
CR → EI	0.116^**^	−0.015, 0.236	−0.011, 0.241	0.001
**Standardized indirect effects**				
CR → ESE → EI	0.069^**^	0.025, 0.124	0.021, 0.120	0.001
Standardized total effects	0.185^**^	0.043, 0.316	0.048, 0.319	0.001

*EE, Entrepreneurial education; EI, Entrepreneurial intention; CR, Creativity; EM, Entrepreneurial mindset; ESE, Entrepreneurial self-efficacy; S.E, Standard error*.

*Significant*

** *p < 0.001*.

## Discussion

Concerning H1, the result indicates that entrepreneurial education positively impacts the entrepreneurial mindset of students. The result of this study is in line with prior researchers (Cui et al., 2019; Handayati et al., 2020) who found that entrepreneurial education provides basic knowledge of entrepreneurship that makes students capable and experts in the new business startup process. The results provide new insights into Chinese entrepreneurial education which helps business students to get knowledge and experience on how to start and manage a new business. Entrepreneurial education enables students that how to identify and exploit entrepreneurial opportunities in the market. It stimulates students to have greater information, knowledge, skills, and encouragement in supporting their entrepreneurial mindset to become entrepreneurs (Yuan et al., 2021). Entrepreneurial education enables the entrepreneurial mindset of the student to have a better perception of numerous results that are crucial for entrepreneurial startups. Moreover, this study suggested that entrepreneurial education affects the entrepreneurial mindset of the student to gain knowledge regarding entrepreneurship and guide them into a good career choice. Furthermore, this study finding is also in agreement with existing literature by Western scholars (Nowiński et al., 2019; Saptono et al., 2020) who believed that entrepreneurial education significantly influenced the entrepreneurial mindset of the student to manage valuable assets and resources for a new venture.

Regarding H2, the findings present that entrepreneurial education had a positive and significant influence on entrepreneurial intention. This finding is similar to previous scholars in the context of Western studies (Westhead and Solesvik, 2016; Sun et al., 2017) who argued that entrepreneurial education effectively drives the entrepreneurial intention of students to become entrepreneurs. Moreover, in the context of the entrepreneurial culture of China, universities allow students to interact with successful entrepreneurs to get some innovative ideas regarding the new business startups. Entrepreneurial motivation from teachers and peers is essential for students in shaping their entrepreneurial intention (Barba-Sánchez and Atienza-Sahuquillo, 2018).

Concerning H3, the results illustrate that entrepreneurial mindset had a positive and significant impact on the entrepreneurial intention of students. This result is similar to prior researchers and noted that students with a higher level of entrepreneurial mindset are more likely to have knowledge, skills, and experience on how to initiate and run a new business (Benchrifa et al., 2017; Burnette et al., 2020). This finding also supports the theoretical contribution of social cognitive theory (Bandura, 1985) which argued that the relationship between cognition factors such as mindset and environmental are positively associated with the entrepreneurial intentions of the student. Social cognitive theory developing an entrepreneurial mindset among students and stimulates their cognitive factors that ultimately lead toward entrepreneurial action (Yuan et al., 2020). The entrepreneurial mindset is shaped by entrepreneurial education and its activities in the school which in turn affects student behavior to become an entrepreneur.

Regarding H4, we found that creativity had a positive and significant effect on entrepreneurial intention. This finding is in line with several previous studies (Hu et al., 2018; Anjum et al., 2021), which remarked that individuals with a high level of creative minds are more likely to peruse a career in entrepreneurship. Creativity is all about something new and innovative and individuals who have creative minds are more capable to articulate innovative ideas into a reality that ultimately leads toward the entrepreneurial intention. Therefore, creativity can be regarded as a valuable factor possessed by individuals, which can stimulate the development of entrepreneurial intention among Chinese students by enhancing the awareness and abilities regarding entrepreneurship, such as opportunity, identification, and exploitation.

Concerning H5, H5a, H5b, and H5c, the results reveal that entrepreneurial self-efficacy positively mediates the relationship between entrepreneurial education, entrepreneurial mindset, and creativity on entrepreneurial intention. This finding is in agreement with prior researchers (Yang, 2014; Wardana et al., 2020). The results suggest that university management facilitates students regarding entrepreneurship education and makes them skillful in handling business activities as well as developing an entrepreneurial atmosphere that ultimately leads to entrepreneurial self-efficacy. Therefore, individuals who perceived a higher level of entrepreneurial self-efficacy are easily identified an opportunity, making an entrepreneurial mindset and think more creatively to commercialize new ideas in the form of product development.

## Conclusion

This study examined the influence of entrepreneurial factors such as education, mindset, and creativity on entrepreneurial intention. This study provides new insights into the context of the Chinese student sample and examined their entrepreneurial intention. This study used SPSS and AMOS software to measure the proposed structural equation model based on 365 valid responses from business students in China. The findings of this study indicated that entrepreneurial mindset has a stronger influence on entrepreneurial intention than entrepreneurial education and creativity. These results show that entrepreneurial self-efficacy positively mediates the relationship between entrepreneurial education, entrepreneurial mindset, and creativity toward entrepreneurial intention.

## Implications and Limitations

Based on the study findings, we offered some practical suggestions for educators and policymakers. First, educators improve their ability and competence particularly regarding entrepreneurship courses, such as in-house training, attend webinars on entrepreneurship, and offer an entrepreneurship certification program. Second, university top leadership develops an entrepreneurial mindset among educators and boosts their confidence to continue their higher studies for a greater outcome. Moreover, for the enhancement of entrepreneurship, university management needs to change the syllabus of entrepreneurship courses through field expertise rather than focus on classroom teaching. Third, the university provides basic facilities to students for entrepreneurial startups, including business incubation centers and other financial supports.

Universities need to support students in developing an entrepreneurial mindset to become entrepreneurs. Fourth, the university could continue to enhance the quality of entrepreneurial education by expanding the teaching materials used on entrepreneurship courses to cultivate the creativity of the student. This would promote a wide range of different learning experiences, not only focused on classroom teaching methods but also developing extra entrepreneurship curriculum activities, which are particularly successful in forming the entrepreneurial intention in the mindsets of students in the Chinese context. Finally, the government should create a better entrepreneurial environment for university students such as setting up a social entrepreneurship support program, providing business capital, and providing free business places where they can easily start their new businesses.

This study provides some limitations that would be considered for future research opportunities. First, data were gathered from Jiangsu and Zhejiang province university students of China, representing a small sample size. The target population was focused only on business department university students. Future research might consider other provinces of China or other students of the country such as vocational schools, IT, and engineering students, and enlarge the sample size to generalize the results. Second, the nature of this study was a cross-sectional design, and data was gathered through a self-administered questionnaire. Future research could conduct on the impact of entrepreneurship education and creativity using entrepreneurial alertness as a mediator among university students with the help of longitudinal research design to add more contribution in the field of entrepreneurship. Further studies also needs to examine the influence of entrepreneurial education and entrepreneurial mindset using creativity and TPB as mediators and extend this entrepreneurial intention model to measure actual entrepreneurial behavior.

## Data Availability Statement

The raw data supporting the conclusions of this article will be made available by the authors, without undue reservation.

## Ethics Statement

The studies involving human participants were reviewed and approved by Ethics Committee of the Jiangsu University China. Written informed consent to participate in this study was provided by the participants. Written informed consent for participation was not required for this study in accordance with the national legislation and the institutional requirements.

## Author Contributions

WJ and MM proposed the research, analyzed the experimental results, and wrote the manuscript. FB and FM designed, carried out the experiments. MT and MR extensively edited and revised the manuscript. All the authors contributed to the article and approved the submitted version.

## Conflict of Interest

The authors declare that the research was conducted in the absence of any commercial or financial relationships that could be construed as a potential conflict of interest.

## Publisher's Note

All claims expressed in this article are solely those of the authors and do not necessarily represent those of their affiliated organizations, or those of the publisher, the editors and the reviewers. Any product that may be evaluated in this article, or claim that may be made by its manufacturer, is not guaranteed or endorsed by the publisher.
